# Application of ERCP in biliary and pancreatic diseases in children: a retrospective analysis of 4-year clinical data from a single center

**DOI:** 10.3389/fped.2025.1722929

**Published:** 2026-01-14

**Authors:** Xiumin Qin, Feihong Yu, Hui Guo, Chunna Zhao, Jie Wu

**Affiliations:** Department of Gastroenterology, Beijing Children’s Hospital, Capital Medical University, National Center for Children’s Health, Beijing, China

**Keywords:** biliary diseases, pancreatic diseases, endoscopic retrograde cholangiopancreatography (ERCP), pediatric, complication, success rate

## Abstract

**Objective:**

This study aimed to summarize the clinical experience of Endoscopic Retrograde Cholangiopancreatography (ERCP) in a pediatric population, analyzing the disease spectrum, procedural characteristics, and clinical outcomes to contribute to the understanding of its application in children.

**Methods:**

This study reviewed the clinical data of children who presented to our hospital and underwent ERCP and related procedures between January 2021 and December 2024. Collected data encompassed patient demographics, specific disease indications, detailed endoscopic techniques employed, procedural success rates, and the incidence and management of related complications.

**Results:**

The study cohort had a mean age of 8.31 ± 3.63 years. The primary indication for ERCP was pancreatic disease, accounting for 72% (134/186) of procedures. These included chronic pancreatitis (*n* = 75), pancreatic trauma (*n* = 17), and acute pancreatitis with pseudocysts (*n* = 17). Biliary diseases constituted 28% (52/186), mainly choledocholithiasis (*n* = 33) and pancreatobiliary maljunction (*n* = 9). Commonly performed endoscopic interventions were pancreatic duct stent placement (*n* = 95), biliary stent placement (*n* = 50), and stone extraction from both ducts (*n* = 70 and 33, respectively). The overall procedural success rate was 90.5% (171/186). A significant difference was noted when stratified by operation time: procedures completed within 60 min had a 96.7% (115/119) success rate, compared to 80.0% (56/70) for those lasting 60 min or longer (*P* < 0.001). Post-procedure complications were recorded in 11 cases (5.9%), including post-pancreatitis (*n* = 6), infection (*n* = 4), and gastrointestinal bleeding (*n* = 1).All complications were all resolved with conservative medical management.

**Conclusion:**

This study confirms that pancreatic diseases (accounting for 72%) are the main indication. Endoscopic Retrograde Cholangiopancreatography (ERCP) has a high success rate (90.5%) in the diagnosis and treatment of biliary and pancreatic diseases in children. However, prolonged procedure time (>60 min) significantly reduces the success rate.

## 1 Background

With the rapid advancement of minimally invasive techniques, endoscopic retrograde cholangiopancreatography (ERCP) has evolved into a pivotal modality for the diagnosis and management of pancreatobiliary diseases in the pediatric population (1). However, due to the distinct anatomical characteristics and technical challenges associated with this demographic, clinical data pertaining to pediatric ERCP remain relatively limited. This study aims to systematically review the application of ERCP in pediatric patients through a retrospective analysis of clinical data spanning four years from a single center, thereby providing valuable evidence to inform clinical practice.

## 2 Materials and methods

### 2.1 Study population

This study retrospectively analyzed the clinical data of 143 pediatric patients who underwent ERCP and related procedures at Beijing Children's Hospital between January 2021 and December 2024, encompassing a total of 186 ERCP procedures. The inclusion criteria were strictly defined as follows: ① age ≤ 18 years; ② presence of clear indications for interventional procedures targeting pancreatobiliary diseases; and ③ availability of complete ERCP procedural records. The exclusion criteria included: ① concomitant severe coagulation disorders; ② presence of absolute contraindications to endoscopy; and ③ cases with incomplete medical records; ④ The patient should undergo outpatient follow-up within 1 year. The study protocol was approved by the Hospital's Ethics Committee (Ethic No. [2025]-E-152-R), and written informed consent was obtained from the parents or legal guardians of all participants.

### 2.2 Procedural technique

All ERCP procedures were performed by experienced endoscopists using an Olympus duodenoscope. Specifically, each operator had previously completed more than 50 pediatric ERCP cases. Following a 12-hour fasting period, all patients underwent the procedure under general anesthesia with endotracheal intubation in the prone position. A Japanese Olympus TJF-260 electronic duodenoscope was employed for all endoscopic interventions.

The duodenoscopic examination and related therapeutic maneuvers were conducted in accordance with established expert consensus guidelines (2). Intraoperative assessment was performed to evaluate for any developmental anomalies of the pancreatobiliary anatomy. Key procedural parameters, including the total procedure time and specific techniques employed, were meticulously documented.

Appropriate ERCP-based therapies were administered based on the underlying pancreatobiliary pathology:

(1) For choledocholithiasis or pancreatic duct stones, procedures such as endoscopic sphincterotomy (EST), endoscopic papillary balloon dilation (EPBD), and stone extraction were performed.
(2) For biliary strictures, balloon or bougie dilation was undertaken, followed by endoscopic retrograde biliary drainage (ERBD) with stent placement.For chronic pancreatitis accompanied by pancreatic duct strictures, endoscopic retrograde pancreatic drainage (ERPD) with stent insertion was conducted.
(3) For acute cholangitis, endoscopic nasobiliary drainage (ENBD) was established.
(4) For patients with pancreas divisum, a series of procedures was carried out, including minor papilla cannulation, pancreatography, minor papillotomy, pancreatic duct dilation, and endoscopic naso-pancreatic drainage (ENPD) where indicated.

### 2.3 Study objectives

The primary objectives of this study were: (1) to analyze the distribution characteristics of indications for pediatric ERCP, and (2) to evaluate the correlation between operative time and success rate. The secondary objectives was to assess the safety of ERCP.

#### 2.3.1 To analyze the distribution characteristics of indications for pediatric ERCP

Based on clinical presentations and intraoperative ERCP findings, the indications for ERCP were categorized into biliary diseases and pancreatic diseases. Biliary diseases were further classified into conditions such as choledocholithiasis, pancreatobiliary maljunction, biliary tract injury, gallstone pancreatitis, and primary sclerosing cholangitis. Pancreatic diseases were subclassified into chronic pancreatitis, pancreatobiliary maljunction, acute pancreatitis with pancreatic pseudocyst, pancreatic trauma, pancreatic duct injury, pancreatic tumors, and inflammatory pancreatic lesions. The number of procedures performed for each disease entity was recorded.

#### 2.3.2 To evaluate factors associated with procedure time and success rate

Technical success was defined as successful duodenal papillary intubation and completion of endoscopic procedures; Clinical success was defined as follow-up of the child's clinical symptoms based on technical success, including symptom relief, reduction in disease recurrence frequency, or improvement in nutritional status (BMI). Demographic characteristics (age, weight, gender), disease type (subcategories of pancreatic/biliary diseases), and procedural parameters (procedure duration, cannulation success rate, complications) were documented. These variables were further analyzed to identify factors correlated with procedure time and technical success rate.

#### 2.3.3 To assess the safety of ERCP: we document the incidence of postoperative complications

Complications were defined and recorded as follows:

(1) Post-ERCP Pancreatitis (PEP): Defined as new or worsened abdominal pain occurring after the procedure, accompanied by a serum amylase level exceeding three times the upper limit of normal at 24 h post-ERCP. Asymptomatic hyperamylasemia was defined as an elevated serum amylase level (>3 times the upper limit of normal) in the absence of abdominal pain.
(2) Bleeding: Categorized as immediate (occurring during or immediately after the procedure) or delayed (occurring hours to weeks post-procedure), primarily related to sphincterotomy. Clinically significant bleeding was defined as a post-procedure decrease in hemoglobin level by ≥3 g/dL compared to the pre-procedure baseline.
(3) Perforation: Suspected in cases of worsening abdominal pain post-ERCP and confirmed by imaging findings such as free air under the diaphragm or in the retroperitoneum on plain abdominal radiograph or computed tomography (CT), or subcutaneous emphysema in the neck. Guidewire-related perforation was a primary concern.
(4) Post-ERCP Cholangitis and/or Cholecystitis: Diagnosed based on the emergence of symptoms such as abdominal pain, fever, and a significant rise in white blood cell count following the procedure.

#### 2.3.4 Statistical analysis

Statistical analyses were performed using SPSS software (version 26.0). Continuous variables with a normal distribution were presented as mean ± standard deviation (Mean ± SD) and compared using the Student's *t*-test. Non-normally distributed continuous data were expressed as median (range) [M (Min, Max)] and compared using non-parametric tests. Spearman's rank correlation coefficient was used for correlation analysis. Categorical data were described as frequencies (percentages) and compared using the Chi-square test or Fisher's exact test, as appropriate. A two-tailed *P*-value of < 0.05 was considered statistically significant.

## 3 Results

### 3.1 Baseline characteristics

A total of 143 pediatric patients were included in this study. The cohort consisted of 66 males (46.2%) and 77 females (53.8%). The age range was from 9 months to 15 years, with a mean age of 8.31 ± 3.630 years. The median body weight was 26.25 kg [interquartile range (IQR): 17.58–41.13 kg], with an overall range of 8.7–107.5 kg.

### 3.2 Spectrum of diseases managed by ERCP

A total of 186 ERCP procedures were included in the analysis. Pancreatic diseases constituted the primary indication, accounting for 134 procedures (72.0%), while biliary diseases were less common, comprising 52 procedures (28.0%). Among the biliary diseases, choledocholithiasis was the most frequent indication (33 procedures, 63.5% of biliary cases), followed by pancreatobiliary maljunction (9 procedures, 17.3%) and postoperative biliary injury (8 procedures, 15.4%). Within the spectrum of pancreatic diseases, chronic pancreatitis was the leading indication (75 procedures, 56.0% of pancreatic cases). The etiologies of chronic pancreatitis included hereditary pancreatitis (44 procedures), pancreas divisum (11 procedures), annular pancreas (3 procedures), and idiopathic causes (11 procedures). Acute pancreatitis with pseudocyst and pancreatic trauma each accounted for 17 procedures (12.7% each). Additionally, ERCP was performed for pancreatitis secondary to pancreatobiliary maljunction (5 procedures), pancreatic tumors (15 procedures), and inflammatory pancreatic lesions (4 procedures).Details are presented in Table 1.

**Table 1:** Spectrum of indications for ERCP procedures.

Disease category	Specific disease	Number of procedures (*n*)	Proportion (%)
Biliary diseases		52	28.0
	Choledocholithiasis	33	17.7
	Pancreatobiliary maljunction	9	4.8
	Postoperative bile duct injury	8	4.3
	Other biliary diseasesa^a^	2	1.1
Pancreatic diseases		134	72.0
	Chronic pancreatitis	75	40.3
	- Hereditary pancreatitis	44	23.7
	- Pancreas divisum	11	5.9
	- Annular pancreas	3	1.6
	- Idiopathic Pancreatitis	11	5.9
	Acute pancreatitis with pseudocyst	17	9.1
	Pancreatic trauma	17	9.1
	Pancreatic tumors	15	8.1
	Pancreatitis due to PBM	5	2.7
	Inflammatory pancreatic lesions	4	2.2
	Other pancreatic diseasesb^b^	1	0.5
Total		186	100.0

PBM, pancreatobiliary maljunction.

a Other biliary diseases: including gallstone pancreatitis (*n* = 1) and primary sclerosing cholangitis (*n* = 1).

b Other pancreatic diseases: pancreatitis associated with ulcerative colitis (*n* = 1).

### 3.3 ERCP and related therapeutic procedures

An analysis of the therapeutic interventions performed during ERCP revealed the following: Endoscopic sphincterotomy (EST) was conducted in 53 procedures. In the context of biliary diseases:Endoscopic stone extraction from the common bile duct was performed in 33 procedures. Endoscopic biliary stent placement was carried out in 50 procedures. Biliary stent exchange was undertaken in 2 procedures. For pancreatic diseases: Pancreatic duct stone extraction was performed in 70 procedures. Pancreatic duct stent placement was conducted in 95 procedures.Pancreatic duct stent exchange was undertaken in 19 procedures.Endoscopic dilation of pancreatic duct strictures was performed in 19 procedures.

### 3.4 Procedural outcomes

This study enrolled 189 ERCP procedures, achieving a total technical success rate of 90.5% (171/189). Procedure duration was the key determinant of technical success: the ≤60-minute group (119 cases) demonstrated a success rate of 96.7% (115/119), significantly higher than the>60-minute group (70 cases) with 80.0% (56/70). The absolute risk difference was 16.7% (95% CI: 8.1%–25.3%), with a relative risk of 1.21 (95% CI:1.09–1.34), showing a statistically significant intergroup difference (*χ*^2^ = 28.324, *P* < 0.001). Furthermore, technical success rates showed no significant correlation with patient age, body weight, or disease classification (*P* > 0.05).

### 3.5 Procedure-Related complications

Post-procedural complications occurred in 11 cases (5.9%). The most common complications included post-ERCP pancreatitis (PEP) in 6 cases (3.2%), infection in 4 cases (2.2%), and gastrointestinal bleeding in 1 case (0.5%). But there were hyperamylasemia in 56 cases (30.1%). All complications were managed conservatively with a full recovery.

## 4 Discussion

Endoscopic retrograde cholangiopancreatography (ERCP) is a well-established procedure for the diagnosis and treatment of pancreatobiliary diseases in adults. However, its application in the pediatric population remains technically challenging. Children are not merely “small adults”; significant differences exist in disease spectrum, anatomical and physiological characteristics, and procedural approaches compared to adults (3).

According to current national and international data, ERCP in adults is primarily utilized for hepatobiliary diseases (2). In contrast, our study demonstrates that pancreatic diseases (72.0%) constitute the primary indication for ERCP in children, challenging the conventional perception dominated by biliary disorders (3, 4). This distribution is highly consistent with recent international multicenter studies, indicating that ERCP has evolved into a pivotal therapeutic modality for pancreatic diseases in the pediatric population (5, 6). Among pediatric pancreatic diseases, chronic pancreatitis (CP) was the most frequent indication (40.3%), with hereditary pancreatitis being the leading etiology for CP (23.7%), consistent with findings from the INSPPIRE consortium (5). Pancreatic ductal anatomic anomalies represent another important etiological category for CP in children (7). In our cohort, pancreas divisum (PD) accounted for 14.7% of cases, aligning with existing literature identifying PD as a contributor to pediatric CP (7). However, the causal relationship between PD and pancreatitis remains controversial (7).Minor papilla sphincterotomy may be considered for patients with idiopathic recurrent acute pancreatitis (iRAP) and PD. Nevertheless, current evidence is predominantly derived from observational studies with inherent limitations. Recent investigations suggest that endoscopic therapy provides limited benefit for pain relief in patients with chronic abdominal pain or CP associated with PD and is not routinely recommended (7). Consequently, close follow-up is essential for children with PD to monitor for recurrent pancreatitis or progression to CP.

With advancements in imaging technology, non-invasive modalities are now primarily used for diagnosing pancreatobiliary diseases, while ERCP is predominantly reserved for therapeutic purposes (2). Although various ERCP-based techniques exist, the primary goal in treating pediatric pancreatobiliary diseases is to improve drainage of the biliary and pancreatic ducts. In our study, for pancreatic diseases, interventions aimed to relieve obstruction, reduce pancreatic duct pressure, alleviate pain, and slow disease progression. Corresponding procedures included pancreatic duct stone extraction (70 sessions), endoscopic retrograde pancreatic drainage (ERPD) with stent placement (95 sessions), and pancreatic duct stricture dilation (19 sessions). The predominance of stone extraction and stent placement reflects a preference for simpler techniques to achieve therapeutic goals (5).Another distinction from adult ERCP practice is the greater emphasis on preserving sphincter of Oddi function during stone extraction from the bile or pancreatic ducts (8). Among the 103 stone extraction procedures in our series, approximately 51.5% involved sphincterotomy, while the remainder utilized endoscopic papillary balloon dilation (EPBD) or temporary stent placement. This demonstrates the feasibility of stone removal in children while striving to preserve papillary function, aligning with the latest European Society of Gastrointestinal Endoscopy (ESGE) guidelines, which recommend balloon dilation over sphincterotomy for stone extraction when possible (9).

Given its technical complexity, the success rate of ERCP is a major concern for clinicians. Our study reported an overall technical success rate of 90.5%, comparable to rates reported in adults. Factors contributing to ERCP failure include anatomical and technical aspects (10). Technical factors primarily relate to the endoscopist's skill and device selection, while anatomical challenges encompass papillary anatomical variants, post-surgical alterations, and strictures or tortuosity of the bile/pancreatic ducts. Unlike adults, children present a smaller working space, developing pancreatobiliary systems, and a distinctly different disease spectrum (11). Nevertheless, our findings indicate that pediatric ERCP success rates can be equivalent to those in adults. Furthermore, our analysis revealed no significant correlation between technical success and patient age or body weight. However, a strong negative correlation was identified between procedural success and duration (*P* < 0.001). The success rate was significantly higher in procedures lasting ≤60 min (96.7%) compared to those exceeding 60 min (80.0%). This suggests that for pediatric ERCP, if the procedure extends beyond 60 min, consideration should be given to whether to continue. While average procedural duration data for adult ERCP is scarce for direct comparison, the median time in our cohort was 55 min, ranging from 20 to 140 min. The significant difference in success rates between the ≤60-minute and >60-minute groups led us to choose 60 min as the cutoff for analysis. A procedure time exceeding 60 min was an independent risk factor for decreased technical success (96.7% vs. 80.0%). This critical threshold holds significant clinical value. Prolonged procedure time often indirectly indicates difficult cannulation (defined as a time to selective cannulation ≥15 min (9) and may be accompanied by operator fatigue and local tissue edema. Therefore, persisting with conventional methods beyond 60 min may yield diminishing returns, and consideration should be given to escalating the technical approach (e.g., using a pre-cut technique, seeking assistance) or terminating the procedure to avoid increasing the risk of complications (12).

This study has several limitations that should be considered when interpreting the results. First, its single-center retrospective design may introduce selection bias and unmeasured confounding factors, despite our efforts to adjust for available variables. Second**ly**, and more critically, key potential confounders such as case complexity (e.g., anatomical variations, previous interventions) and operator experience were not systematically recorded or analyzed. These factors likely influence both procedural duration and technical success, potentially confounding the observed association between longer operation time and lower success rates. Therefore, the strength of this association should be interpreted with caution. Future prospective studies that rigorously document case difficulty and operator skill level are needed to validate and refine our findings. Thirdly, the relatively limited sample size may affect the statistical power for analyzing rare complications or subgroups. Finally, the lack of long-term follow-up data prevents an assessment of the long-term benefits of the sphincter-preserving strategies.

This study confirms that pediatric ERCP possesses unique characteristics regarding its indications, key factors determining technical success, and therapeutic principles. In clinical practice, while pursuing a high technical success rate, optimizing procedure time and incorporating organ function preservation as a core principle are paramount.

## Data Availability

The original contributions presented in the study are included in the article/Supplementary Material, further inquiries can be directed to the corresponding author.
